# Second International Congress on Chocolate and Cocoa in Medicine Held in Barcelona, Spain, 25–26th September 2015

**DOI:** 10.3390/nu7125502

**Published:** 2015-12-01

**Authors:** Margarida Castell, Sandra Saldaña-Ruíz, Maria José Rodríguez-Lagunas, Àngels Franch, Francisco J. Pérez-Cano

**Affiliations:** 1 Department of Physiology, Faculty of Pharmacy, University of Barcelona, Barcelona 08028, Spain; ssaldana@ub.edu (S.S.-R.); angelsfranch@ub.edu (A.F.); franciscoperez@ub.edu (F.J.P.-C.); 2 Nutrition and Food Safety Research Institute, University of Barcelona, Santa Coloma de Gramenet 08921, Spain; 3 Department of Physiological Sciences II, Faculty of Medicine, University of Barcelona, L’Hospitalet del Llobregat 08907, Spain; mjrodriguez@ub.edu

## 1. Preface

Cocoa powder is a product derived from the beans of the *Theobroma cacao* tree, which is considered a good source of fiber (26%–40%), proteins (15%–20%), carbohydrates (about 15%) and lipids (10%–24%; generally, 10%–12%). It also contains minerals, vitamins and some bioactive compounds such as flavonoids, fiber, and methylxanthines, as is the case of theobromine. In recent years, cocoa and its derivatives such as chocolate have been the focus of increasing interest, mainly because of their high content of flavonoids, which are compounds with antioxidant activity. Cocoa contains flavanols such as (–)-epicatechin and catechin as monomers, and dimers or larger polymers derived from both of these, known as procyanidins. Due to this particular composition, and mainly based on its activity as an antioxidant, as well as through other mechanisms, cocoa consumption has been reported to promote beneficial effects on cardiovascular health, metabolism, brain and immune functions, and in cancer prevention, among others.

In order to further our understanding of, and disseminate the latest findings on the healthy properties of cocoa and chocolate, the International Society of Chocolate and Cocoa in Medicine (ISCHOM) was founded in 2010 in Florence (http://ischom.com/ischom/). This Society aims to gather information and become a forum of discussion and debate on cocoa and chocolate, not only among researchers from around the world, but also to introduce the science involved and the latest findings to the public. Cultural and educational promotion of the benefits of cocoa and chocolate on human health is another of the Society’s major concerns. Finally, ISCHOM provides information on developing healthy habits regarding the inclusion of cocoa and chocolate in our diet.

In this context, after the first congress in Florence in 2014, ISCHOM held its second meeting in Barcelona, Spain, on 25 and 26 September 2015 (https://www.eiseverywhere.com/ehome/117409/263536/). By means of these annual meetings, the Society pursues the constant sharing and updating of current knowledge concerning the health properties of cocoa and chocolate. The event not only brings together international researchers, but also diverse companies in order to strengthen the knowledge in this field. About 90 delegates from 12 different countries attended the Society’s second congress. It was organized into five scientific sessions with two lectures each.

The sessions that took place in Barcelona were focused on different topics in order to shed some light on a wide range of valuable effects of chocolate and cocoa on our health. Some of the issues discussed were the role of chocolate and cocoa as cardioprotective agents and its health claims, their effects on metabolism and their own metabolism; the possible role of cocoa as a preventive therapy for diabetes and allergies; its influence on microbiota; and the beneficial effects of cocoa on the nervous system.

The opening session outlined the scientific thought on this matter spiced with wit. The congress also included a special session focused on heritage and innovation in chocolate that delighted the audience. Furthermore, two discussion sessions of oral communications also took place. The first session related to assessing the intake of cocoa and its effect, the relationship between cocoa flavanols, cognitive performance and cerebral blood flow; and the effect of other bioactive compounds of cocoa, methylxanthines such as theobromine, and their outcomes on rat lymphoid tissues were ascertained. The second oral communication discussion session concerned the assessment of the antioxidant capacity and polyphenol content of different varieties of cocoa, how cocoa intake improves hepatic lipid metabolism in rats, and how flavanols present in cocoa may confer benefits by diminishing brain damage caused by strokes in mice. In addition, the poster session was exhibited during the entire congress showing state-of-the-art research about chocolate and cocoa in medicine for all the attendants.

## 2. Summary of Lectures

### 2.1. Hesitations of an Extraterrestrial Scientist: What is Chocolate?

#### Mans, C.

An alien visiting Earth discovers a strange object on the ground. To identify it he proceeds to carry out various tests and analyses. In his ship, the object undergoes mechanical, thermal and electrical tests, as well as some chemical analyses. The alien notes that it breaks easily, softens at low temperatures, melting completely, while at higher temperatures it decomposes and carbonizes. It is not electrically conductive, not soluble in water or polar solvents, and has not the ordered structure that at first glance it seems to have. Further microscopic analysis allows the identification of an amorphous mixture of at least three different types of substances, one of which is pure and crystalline while the others are complex mixtures. Elemental analysis determines the presence of C, H, O, N, and other elements in trace amounts. The alien gives a small amount of the substance to a dog who subsequently suffers from severe intestinal problems.

Therefore, in the absence of further analysis, the alien tentatively concludes that the substance is a kind of toxic hydrocarbon bitumen of unknown origin and of no industrial interest because of its low melting point. However, more research must take place once the product is transported to his planet.

Chocolate lovers were lucky that no more than a sample was transferred to the alien planet, and not the Earth’s entire production.

### 2.2. Cocoa, Polyphenols and Cardiovascular Disease

#### Estruch, R.

Evidence based on epidemiological studies suggests that dietary flavonoids may play a critical role in the prevention of coronary heart disease (CHD). Cocoa (*Theobroma cacao*) and its derived products, such as chocolate, represent a very rich source of dietary flavonoids, containing a higher content per serving than tea, red wine, legumes or fruits.

The health benefits associated with cocoa consumption have been related to their protective effect mainly against cardiovascular diseases, but also in other diseases such as age-related cognitive decline. Observational studies have shown that the Kuna Indian population from the San Blas Islands of Panama have very low rates of hypertension and cardiovascular disease, effects that have been related to their high intake of cocoa. Clinical trials have also evaluated the effects of cocoa intake on different cardiovascular risk factors. Several studies have confirmed that cocoa intake reduces blood pressure in normotensive and hypertensive subjects. In fact, dark chocolate intake increases nitric oxide (NO) generation, which leads to vasodilatation, and reduces systolic and diastolic blood pressure by 2.77 mmHg and 2.20 mmHg, respectively, whereas white chocolate does not reduce blood pressure. Another mechanism by which flavanols may lower blood pressure is by the inhibition of angiotensin-converting enzyme (ACE). In addition, cocoa intake improves lipid profile and insulin sensibility, reduces platelet activity and function, and ameliorates endothelial dysfunction. At least some of these other beneficial effects have also been attributed to anti-inflammatory and antioxidant activities of polyphenols contained in cocoa.

Therefore, the addition of dark chocolate to a well-balanced healthy diet such as a Mediterranean diet, offers a palatable option to preventing cardiovascular disease.

### 2.3. Current Status of Nutrition and Health Claims in Europe with a Focus on Antioxidants and Chocolate

#### Verhagen, H.

Functional foods are closely associated with claims on foods. There are two categories of claims on foods: nutritional claims and health claims. In the European Union, health claims on (functional) foods and food supplements must be scientifically substantiated. In December 2006, the European Union published its Regulation 1924/2006 on nutrition and health claims made on foods (http://eur-lex.europa.eu/LexUriServ/site/en/oj/2007/l_012/l_01220070118en00030018.pdf).

The European Food Safety Authority (EFSA) provides scientific advice to the European Commission for health claims submitted under Regulation 1924/2006. With regard to general function health claims, in Europe more than 44,000 proposals were made, from which the European Commission prepared a list of about 4600 submitted to EFSA for scientific evaluation.

Hitherto EFSA have evaluated about two-thirds of these, the large majority of which having received the judgment “a cause and effect relationship has not been established”. EFSA has published hundreds of opinions on health claims, some of which are positive, some of which are negative, and a few with insufficient evidence (http://www.efsa.europa.eu/en/nda/ndaclaims.htm; http://ec.europa.eu/food/food/labellingnutrition/claims/index_en.htm).

Regulation No 432/2012 was published on 25 May 2012 (http://eur-lex.europa.eu/LexUriServ/LexUriServ.do?uri=OJ:L:2012:136:0001:0040:EN:PDF). A list of 222 general function health claims has been approved by the Commission. For the remaining *ca.* 1500 claims on botanicals it is not clear if and how they will be evaluated in terms of their scientific substantiation. Now Europe has a list of authorized and non-authorized health claims (http://ec.europa.eu/nuhclaims/).

With regard to antioxidants/oxidative damage (search “oxidat”), EFSA has evaluated almost 200 health claims: a cause and effect relationship was found established and authorized for only eight health claims (vitamins C, E and B2; the minerals copper, manganese, zinc and selenium; olive oil polyphenols standardized by hydroxytyrosol and its derivatives). Regarding “cocoa” and “chocolate”, a total of 12 generic (article 13.1) health claims were found to be not scientifically substantiated and hence were not authorized, but two (article 13.5) health claims on “cocoa flavanols” were found to be scientifically substantiated and subsequently authorized.

Starting 14 December 2012, all claims that are not authorized or on hold/under consideration are prohibited.

### 2.4. Modulation of Obesity-Related Inflammation and Fatty Liver Disease by Cocoa: A Potential Role for the Mitochondria

#### Lambert, J.D.

Fatty liver disease is an important co-morbidity of obesity. Previously, we have reported that dietary cocoa (*Theobroma cacao*) supplementation can reduce obesity-related inflammation and markers of insulin resistance in high fat-fed mice. In the present study, we examined the effect of cocoa supplementation on obesity-related fatty liver disease. Male C57BL/6J were fed a high fat (HF) (60% kcal from fat) diet for eight weeks and then randomized to continue the HF diet or to consume an HF diet supplemented with 80 mg/g cocoa powder (HF→HFC) for an additional 10 weeks. Dietary cocoa reduced hepatic lipid content by 40% and plasma alanine aminotransferase levels by 32% compared to obese mice. Cocoa treatment also reduced the expression of hepatic pro-inflammatory markers (monocyte chemoattractant protein 1 and macrophage inflammatory protein 1a), but increased expression of hepatic markers of alternative (anti-inflammatory) macrophage activation (interleukin 10 and CD163) compared to HF-fed obese controls. Analysis of data related to mitochondrial biogenesis, oxidative stress and antioxidant response showed that cocoa increased mitochondrial biogenesis and increased mitochondrial antioxidant response compared to obese controls. We found cocoa treatment increases hepatic mitochondrial DNA copy number by 48.6% compared to HF-fed mice, suggesting increased mitochondrial biogenesis. Using real-time PCR, we found that mRNA levels of sirtuin (sirt) 1, peroxisome proliferator-activated receptor gamma coactivator 1a, and nuclear respiratory factor 1, genes that regulate mitochondrial biogenesis, were significantly increased in HF→HFC mice compared with HF-fed controls. Cocoa treatment reduced hepatic lipid peroxidation by 57% compared to HF-fed controls, indicating reduced oxidative stress. These effects were related to an increase in the expression of Sirt3, an important mitochondrial redox regulator, as well as the expression and activity of manganese superoxide dismutase and glutathione peroxidase. In summary, our results demonstrate that the beneficial effects of cocoa supplementation on non-alcoholic fatty liver disease may be mediated by increases in mitochondrial biogenesis and related antioxidant response signaling.

Acknowledgements: supported by a grant from The National Center for Complementary and Alternative Medicine (AT004678).

### 2.5. What Can Chocolate and Cocoa Learn from Metabolomics?

#### Garcia-Aloy, M.; Llorach, R.; Urpi-Sarda, M.; Vázquez-Fresno, R.; Jáuregui, O.; Andres-Lacueva, C. *

There is a growing body of evidence of the beneficial cardiovascular effects of cocoa consumption. Untargeted metabolomics is used as a hypothesis-generating tool. The main aim of this work was to contribute to the identification of biomarkers related to food ingestion (biomarkers of intake), as well as their potential association with health (biomarkers of effect) in a population at high-risk of cardiovascular disease, using an untargeted High-Performance Liquid Chromatography coupled to Quadropole Time-of-Flight Mass Spectrometry (HPLC-Q-ToF-MS) metabolomics strategy in acute and short-term clinical trials, as well as in observational studies.

Dietary cocoa fingerprinting was characterized by a complex metabolic pattern linked to cocoa phytochemicals (alkaloids and polyphenols) and processing-derived compounds, as well as endogenous metabolites. A large proportion of metabolites were characteristic of cocoa exposure independently of the study design. They belong both to theobromine metabolism and to microbial-derived metabolism of polyphenols. With respect to the endogenous metabolome, methylglutarylcarnitine showed reduced levels associated with cocoa consumption, both in the short-term clinical trial and in the observational study. Because of the potential role of acylcarnitines in insulin resistance, this observation could provide a mechanistic insight into the beneficial effects of cocoa consumption on insulin sensitivity previously described in epidemiological studies. Finally, to improve the prediction of cocoa consumption, a combined urinary metabolite model was constructed. Receiver operating characteristic (ROC) curves were performed to evaluate the model and individual metabolites. The area under curve values (95% confidence interval) for the model were 95.7% (89.8%–100%) and 92.6% (81.9%–100%) in training and validation sets, respectively, whereas the AUCs for individual metabolites were <90%. Discriminating metabolites of cocoa exposure were replicated in three studies with different designs, increasing the level of evidence from observed associations. Since some of the discriminating compounds are produced by gut microbiota this reinforces the hypothesis that the microbial food metabolome is an important source of dietary biomarkers. The predictive capacity of dietary exposition was improved using multimetabolite combined models compared with the same compounds individually.

Acknowledgement: This work has been supported by MINECO and co-founded by FEDER: AGL2009-13906-C02-01; CONSOLIDER-INGENIO 2010 Program, FUN-C-FOOD (CSD2007-063); and PCIN-2014-133 JPI HDHL_Biomarkers. We also thank the award of 2014SGR1566 from AGAUR. M. Urpi-Sarda thanks the “Ramón y Cajal” program (RYC-2011-09677), and all authors thank MINECO and Fondo Social Europeo.

### 2.6. Could a Cocoa Diet Be Beneficial for Diabetes?

#### Goya, L.

Type 2 diabetes mellitus (T2DM) is a complex metabolic disorder characterized by sustained hyperglycemia that results from defects in insulin secretion, insulin action or a combination of both. Prevalence of T2DM is increasing globally and it has reached epidemic proportions; in fact, diabetes is currently one of the most costly and burdensome chronic diseases (Whiting, D.R., *et al.* *Diabetes Res. Clin. Pract.* 2011, *94*, 311–321). Despite the fact that several drugs are available for the treatment of diabetes, adverse effects and drug resistance are of great concern. Therefore, there is an urgent need to continue working on the prevention and control of this pathology and, as a promising alternative, researchers are seeking natural products to prevent or treat T2DM because of their potential beneficial effects on health and because of their safeness (Williamson, G. *Mol. Nutr. Food Res.* 2013, *57*, 48–57). Cocoa has been shown to exert antidiabetic effects by lowering glucose levels. We have recently shown that cocoa flavanols may have antidiabetic potential *in vitro* by promoting the survival and function of pancreatic beta-cells and improving the insulin signaling pathway in liver cells (Cordero-Herrera, I., *et al.* *Mol. Nutr. Food Res.* 2013, *57*, 974–985; Martín, M.A., *et al.* *Mol. Nutr. Food Res.* 2014, *58*, 447–456). Therefore, we decided to investigate the effects of a cocoa-rich diet in Zucker diabetic fatty (ZDF) rats. Male ZDF rats were fed a control or cocoa-rich diet (10%), and Zucker lean animals received the control diet. The ZDF rats supplemented with cocoa showed a significant decrease in body weight gain, glucose and insulin levels, as well as an improved glucose tolerance. Our results showed that cocoa feeding during the pre-diabetic state reduces insulin resistance and increases beta-cell mass and function in ZDF rats (Fernández-Millán, E., *et al.* *Mol. Nutr. Food Res.* 2015, *59*, 820–824). Cocoa-rich diet further ameliorated the hepatic insulin resistance by favoring insulin signaling, glucose transport, glycolysis and glycogenesis and reducing gluconeogenesis (Cordero-Herrera, I., *et al.* *J. Nutr. Biochem.* 2015, *26*, 704–712). Furthermore, a cocoa-rich diet protects the hepatocytes of ZDF rats by improving their antioxidant competence and by ameliorating diabetes-induced damage to lipid metabolism through multiple signaling pathways (Cordero-Herrera, I., *et al.* *Food Res. Int.* 2015, *69*, 194–201). These findings provide the first *in vivo* evidence that a cocoa-rich diet may delay the loss of functional beta-cell mass and protect liver activity in order to delay the onset of T2DM.

### 2.7. Cocoa in the Prevention of Allergy

#### Castell, M.

Allergy is an adverse response in which the immune system reacts against innocuous agents. This response is produced by the activation of Th2 lymphocytes, which, that by means of cytokines, favor the synthesis of IgE antibodies. IgE is able to bind to a receptor present on the surface of mast cells and cause their degranulation that involves the secretion of mediators, such as histamine and proteases. The release of mediators will eventually generate allergic symptoms such as urticaria, rhinitis, asthma, and, in the worst cases, anaphylactic shock.

There are studies showing the protective effects of flavonoids on allergy (Castell, M., *et al.* *Curr. Pharm. Des.* 2014, *20*, 973–987). Thus, clinical and preclinical studies on respiratory allergy show that flavonoid intake can decrease some respiratory symptoms and the concentration of IgE and histamine in serum. On the other hand, previous studies have shown the influence of cocoa diet on immune response in immunized rats, attenuating cytokines and antibodies related to Th2 immune response (Pérez-Berezo, T., *et al.* *Mol. Nutr. Food Res.* 2009, *53*, 389–397). Taking these results into account, we aimed to establish the immunomodulator effect of cocoa on an allergic reaction induced in rats.

From our results, we saw that allergic rats fed with 10% cocoa diet produced lower amounts of specific IgE and other Th2 antibodies (Abril-Gil, M., *et al.* *Pharmacol. Res.* 2012, *65*, 603–608), and also Th2 cytokines (Abril-Gil, M., *et al.* *J. Nutr. Biochem.* 2015, in press). However, after the induction of an anaphylactic response, the alterations induced in motor activity, body temperature, intestinal permeability and hematocrit were not affected by the diet. Nevertheless, when we determined the protease released from mast cells, which was much higher in allergic animals, the cocoa diet greatly prevented this increase. Moreover, the cocoa intake produced the downregulation of the gene expression of mast cell IgE receptor and protease in the intestine. In order to understand the role of cocoa flavonoids in its anti-allergic ability, we compared the effect of a conventional cocoa diet with a diet containing purer cocoa flavonoids. Results showed that there may be cocoa compounds other than flavonoids that enhance cocoa’s anti-allergic effect (Abril-Gil, M., *et al.* *J. Nutr. Biochem.* 2015, in press).

In conclusion, cocoa, by means of its flavonoids and mainly by other compounds, has the potential to suppress the Th2 immune response and also to attenuate the release of mast cell mediators, but these effects are not enough to completely protect against an anaphylactic response.

Acknowledgments: Grants AGL2008-02790 and AGL2011-24279 from the Spanish Ministry of Science and Innovation and the Ministry of Economy and Competitiveness, respectively.

### 2.8. Interaction of Cocoa Polyphenols with Gut Microbiota: Potential Health Effects in Humans

#### Tomás-Barberán, F.A.

Polyphenols present in cocoa and their health effects have been the subject of active research over the last 25 years. The physiological relevance of the clinical trials has, however, been rather limited due to the large inter-individual variability observed. The absorption of these phytochemicals in the gastrointestinal tract is limited and they reach the colon almost unaltered where they interact with the colon microbiota. The colon microorganisms have a two-way relationship with cocoa polyphenols, as on the one hand these phytochemicals modulate the microbiota population, while on the other the microbiota transform polyphenols producing metabolites that differ from the original cocoa constituents. Cocoa polyphenols activate the development of some bacterial groups while inhibiting the growth of others. This may be associated with some health benefits. Colonic microbes can metabolize cocoa proanthocyanidins, leading to metabolites that are better absorbed than the original compounds or can provide other health effects. Therefore, depending on the composition of the gut microbiome, the bioavailability and biological effects of cocoa polyphenols can be modulated. The identification of the bacteria responsible for the metabolic transformation of specific phenolics is an active area of research, and members of the Coriobacteriaceae and different Lactobacilli and Bifidobacteria have been associated with specific metabolic transformations of proanthocyanidins in the gut. The mechanisms through which these microbiota metabolites exert their biological effects are currently being studied. This means that individuals can produce, absorb and excrete different proanthocyanidin metabolites, and therefore enjoy different biological effects due to cocoa intake, depending on their microbiome, and this could partly explain the inter-individual variability observed in human intervention studies with cocoa products. This opens new opportunities for the development of cocoa-based nutraceuticals and functional foods. The discovery of the human enterotypes will eventually have future implications in nutritional and medicinal plant treatments and in the development of specific drugs and food products for individuals with a specific enterotype within the field of personalized nutrition.

### 2.9. Cocoa Modifies Interaction of Microbiota with Intestinal Immune System

#### Pérez-Cano, F.J.

Crosstalk is the term used for designing the interaction between host cells and microbes. Among other mechanisms, this takes place when certain molecules of the microorganisms are recognized by the toll-like receptors (TLRs) in the body cells, mainly at the mucosal level. TLRs belong to the pattern-recognition receptors and represent the first line of defense against pathogens, playing a pivotal role in both innate and adaptive immunity. Dysregulation in the activity of such receptors can lead to the development of chronic and severe inflammation as well as immunological disorders (Ospelt, C., *et al.* *Int. J. Biochem. Cell Biol.* 2010, *42*, 495–505).

Several *in vitro*, *in vivo* and clinical approaches have demonstrated that components present in the diet, are able to modulate TLR-mediated signaling pathways (Pérez-Cano, F., *et al.* *Antioxidants* 2014, *3*, 649–670). Among these dietary factors, we can find polyphenols, and, in this context, certain studies have demonstrated how cocoa, as a rich source of flavonoids, is able to modify this type of interaction. In particular, cocoa flavonoids and cocoa fiber have shown their ability to modify the composition of the microbiota and to modulate TLR gene expression (Massot-Cladera, M., *et al.* *Arch. Biochem. Biophys.* 2012, *527*, 105–112; Massot-Cladera, M., *et al.* *Br. J. Nutr.* 2014, *112*, 1944–1954; Massot-Cladera, M., *et al.* *J. Funct. Foods* 2015, *19*, 341–352). Moreover, flavonoids present in cocoa have demonstrated the ability to regulate the downstream signaling molecules involved in the TLR pathway.

Due to this modulatory action, among others, cocoa influences the immune system, particularly the inflammatory innate response and the systemic and intestinal adaptive immune response. To date, a cocoa diet is able to modulate pro-inflammatory cytokines, to change the lymphocyte composition and functionality of adaptive response (by means of inhibiting Th2 cell function) and to change innate immunity (potentiating certain aspects of mucosal Natural Killer response). However, further research should be directed to elucidate the cocoa compounds involved in such effects and also the possible medical approaches to these impacts (Pérez-Cano, F.J., *et al.* *Front. Pharmacol.* 2013, *4*, 71).

Overall, although the molecular targets involved in the modulatory action of cocoa on TLR-mediated signaling pathways, and its consequences, are not fully understood, some synergistic mechanisms of cocoa compounds are suggested for the preventive effect on certain inflammatory, immune-mediated or chronic diseases.

Acknowledgments: Grants AGL2008-02790 and AGL2011-24279 from the Spanish Ministry of Science and Innovation and the Ministry of Economy and Competitiveness, respectively.

### 2.10. Cocoa Flavonoids and Brain Health: Physiological and Molecular Mechanisms Underpinning Their Beneficial Effects

#### Vauzour, D.

Accumulating evidence suggests that diet and lifestyle can play an important role in delaying the onset or halting the progression of age-related health disorders and in improving cognitive function. A growing number of dietary intervention studies in humans and animals, and in particular those using cocoa flavonoids, have been proposed to exert a multiplicity of neuroprotective actions within the brain, including the potential to protect neurons against injury induced by neurotoxins, the ability to suppress neuroinflammation and the potential to promote memory, learning, and cognitive functions. These effects appear to be underpinned by two common processes. First, they are capable of interactions with critical protein and lipid kinase signaling cascades in the brain, leading to an inhibition of apoptosis triggered by neurotoxic species and to a promotion of neuronal survival and synaptic plasticity. Second, they induce beneficial effects on the vascular system, leading to changes in cerebrovascular blood flow capable of causing enhanced vascularization and neurogenesis, two events important in the maintenance of cognitive performances. Together, these processes act to maintain brain homeostasis and play important roles in neuronal stress adaptation and thus cocoa flavonoids might have the potential to prevent the progression of neurodegenerative pathologies.

### 2.11. The Chocolate Way Project, Candidate for the European Cultural Route

#### Tresserras, J.

The Association “The Chocolate Way” intends to pursue the following main objectives:

(a) To promote, enhance and protect the European artistic, historic, and cultural heritage, material and immaterial, linked to cocoa and chocolate in Europe over the centuries, as well as expanding its knowableness by putting Chocolate Routes onto a network, highlighting both the role that chocolate has had over the centuries and its present role fostering communication between European populations and distant cultures, even overseas, and bringing them closer through trade.

(b) To promote the production of artisan-made chocolate as a symbol of European identity, through the diffusion of craft knowledge and of traditional techniques, promoting the construction of a common European citizenship and contributing, more generally, to improving the spread of the European image and cultural identity.

(c) To enhance and promote the artisan confectionery product, in particular chocolate, as part of a healthy Mediterranean diet with the nutritional values ascribable to it.

(d) To promote the official recognition of the Chocolate Way as a European Cultural Route of the Council of Europe and to create synergies with the Cocoa Route promoted by UNESCO in Latin America and the Caribbean.

(e) To promote and support projects of cooperation in cocoa’s origin countries to assure a better tenability of the entire chocolate supply chain.

An example of sites and experiences related to cocoa and chocolate history, culture and creativity in Barcelona and Catalonia are now articulated to reinforce the creation of a regional node of this transnational route. Initiatives such as CHIELI—Heritage in European Life and Identity—financed by the European Union, through to the COSME Program (2014–2020) and the declaration of Catalonia as a European Region of Gastronomy for 2016 will contribute to achieve this.

### 2.12. Physico-Chemical Characterization of New Chocolate Textures

#### Bayés-García, L.; Calvet, T. *; Cuevas-Diarte, M.A.; Rovira, E.; Sato, K.; Ueno, S.

Chocolate is made up of cocoa butter (CB) crystals as a continuous body, in which tiny particles of sugar, cacao mass, and other ingredients are dispersed. Sharp melting and a quick release of flavor and sweetness/bitterness are determined by the melting behavior of CB crystals. Cocoa butter exhibits six different polymorphic forms, referred to as I–VI. Among them, form V is industrially promoted through tempering processes, as this polymorph provides the desired melting, textural, and mouth-feel characteristics of chocolate. Dynamic thermal treatments are highly significant in obtaining specific polymorphic forms of lipids. In this study, we examined the influences of dynamic thermal treatments on the development of new textures of chocolate revealing a soft mouth-feel (velvet effect), which can be obtained by tailoring rapid cooling and subsequent heating treatments. Fluidized chocolate (tempered mixture of chocolate and cocoa butter) was sprayed on two different substrates (metal and tempered chocolate) at different substrate temperatures (4 °C, 12 °C, 16 °C and 18 °C). Polymorphic crystallization and transformation was monitored *in situ* by using laboratory-scale X-ray diffraction. Thermal treatments enabled the formation of thin layers of cocoa butter crystals with much smaller particle sizes and a low melting point compared to normally tempered chocolate, leading to the creation of a soft mouth-feeling. The results confirmed the template effect caused by the substrate of chocolate, as polymorphic transformations occurred more rapidly than metallic substrates.

## 3. Selected Abstracts

### 3.1. Acute Effect of Cocoa Flavanol Intake on Cognitive Performance and Cerebral Blood Flow in Rest and Following Exercise in Well-Trained Athletes

#### Decroix, L. *; Tonoli, C.; Dias-Soares, D.; Heyman, E.; Tagougui, S.; Meeusen, R.

Background: Both exercise and cocoa flavanols (CF) can acutely improve cognition function. Increased brain perfusion is a mechanism causing improved cognitive function after CF and exercise. Objectives: To study the effects of acute CF intake on exercise-induced alterations in executive cognitive function and (prefrontal) cerebral blood flow (CBF) in well-trained men.

Methods: Twelve healthy, well-trained men (VO_2_max 63 mL/kg/min) participated in this randomized, double-blind crossover intervention trial. Participants consumed a 903.75 mg CF-chocolate drink or placebo chocolate drink (PL), immediately followed by a baseline cognitive test (CT). A second CT was performed 100 min after consumption of the drink. This was followed by a 30-min time trial. Subsequently, a third CT was performed immediately after the time trial. Prefrontal near-infrared spectroscopy (NIRS) was applied during CTs to measure CBF, *i.e.*, oxygenated, deoxygenated and total hemoglobin (HbO_2_, HHb, and Hbtot, respectively). Reaction times and accuracy on a simple reaction test and stroop task were registered.

Results and Discussion: Repeated measures ANOVA showed a main effect of time for all outcome measures. A significant interaction effect (time × condition) (*p* < 0.05) was found for Hbtot during the stroop test. No main effects of condition were found for any of the outcome measures. ANCOVA showed that, in rest, CF intake significantly increased Hbtot and HbO_2_ during CT compared to PL (*p* < 0.05), however it did not significantly influence cognitive performance nor HHb. After exercise, no significant differences in cognitive performance and CBF between CF and PL were observed. This study shows that acute CF intake increases CBF (Hbtot, HbO_2_) during a CT, but does not result in an improved cognitive performance. Following exercise, there is no additional effect of CF intake on CBF during a CT.

### 3.2. Short Term Effects of Cocoa Theobromine on Primary and Secondary Lymphoid Tissues in Rats

#### Camps-Bossacoma, M. *; Bitlloch-Obiols, M.; Abril-Gil, M.; Franch, A.; Pérez-Cano, F.J.; Castell, M.

Background: Cocoa contains bioactive compounds such as flavonoids, fiber and methylxanthines, mainly theobromine (Katz, D.L., *et al.* *Antioxid, Redox Signal.* 2011, *15*, 2779–2811; Hurst, W.J., *et al.* *Nature* 2002, *418*, 289–290). Although many effects of cocoa have been associated with flavonoids (Massot-Cladera, M., *et al.* *Nutrients* 2013, *5*, 3272–3286), theobromine could also have a role in its effects and this could be reflected in the actions of cocoa on the immune system.

Objective: The aim of the present study was to establish the role of cocoa theobromine, in the short term, on the primary (thymus) and secondary (spleen and lymph nodes) lymphoid tissues in rats.

Methodology: Female Lewis rats (three weeks old) were fed either a reference diet, a diet containing 10% cocoa or a reference diet added with the same amount of theobromine provided by cocoa (0.25%). After seven days of free access to water and chow, thymus, spleen and mesenteric lymph nodes (MLN) were removed to isolate lymphocytes to be immunostained and analyzed by flow cytometry.

Results: In the thymus, lymphocytes were characterized according to the expression of CD4 and CD8 coreceptors. The proportion of CD4^+^CD8^+^ (DP) cells was 95% in the reference group and this percentage decreased in animals fed with cocoa and those fed with theobromine. In addition, both nutritional interventions induced an increase in the proportion of CD4^+^CD8^−^ with no changes in that of CD4^−^CD8^+^ cells. In the spleen, 10% cocoa diet, and also theobromine diet, significantly decreased the TCRγδ^+^ and NK cell proportions. Although total TCRαβ^+^ cell percentage was not modified, there was an increase in the T helper lymphocyte proportion and a decrease in T cytotoxic and NKT cell percentage induced by both cocoa and theobromine diets. In MLN, 10% cocoa and 0.25% theobromine diet induced changes in TCRαβ^+^ subsets: T helper and NKT lymphocyte proportion decreased and, as a consequence, T cytotoxic cells increased. In addition, both dietary interventions increased the proportion of TCRγδ^+^ CD8αα^+^.

Conclusion: These results showed that a 10% cocoa diet and a 0.25% theobromine diet produced the same changes in lymphocyte composition in the thymus, spleen and MLN. Therefore, we can conclude that theobromine is the main cacao compound responsible for the changes in lymphoid tissues.

Acknowledgments: AGL2011-24279 from the Spanish Ministry of Economy and Competitiveness.

### 3.3. Exploring the Nutraceutical Value of Fine or Flavour Trinitario Cacao Varieties: Antioxidant Capacity and Polyphenol Content of 24 Imperial College Selections from Trinidad

#### Pilgrim, S. *; Yen, I.C.; Sukha, D.; Bekele, F.

Cocoa is widely consumed as chocolates and in desserts, and used to produce beverages, cosmetics, pharmaceuticals and toiletries. Recently, it has become recognized for nutraceutical benefits including promotion of cardiovascular health, decreased oxidation of LDL to prevent atherosclerosis formation, reduction in LDL cholesterol and high antioxidant capacity. Cocoa capsules containing high quantities of antioxidants are currently marketed in the health-food industry. Cocoa is now regarded as a functional food or as a medicine. Twenty-four Imperial College Selection (ICS) cacao varieties were thus investigated with a view to their exploitation as a potential source of natural antioxidants due to their phenolic composition and dietary antioxidant potential. These varieties produce fine or flavor Trinitario cocoa beans and are sold in a niche market due to desirable flavor and quality attributes. Some Trinitario cacao varieties are also valued for their favorable agronomic traits such as large seed size and good yield. The anti-oxidant activity, AA (Trolox equivalent antioxidant capacity (TEAC) and vitamin C equivalent antioxidant capacity (VCEAC)) of ethanolic extracts prepared from cocoa beans were measured by two different standards using a similar method of analysis. Folin–Ciocalteu reagent was used to estimate the total polyphenol content (TPP). The TEAC values were within the range of 755 to 7442 µmol/g TE and the VCEAC levels fell within a range of 397 to 3917 µmol/g VCEAC. Total extractable phenolics (TPP) were 59 to 164 mg/g for cocoa beans. Significant differences (*p* < 0.05) were observed for AA and TPP. The varieties with high AA and TPP can be exploited for their nutraceutical value in the production of dark chocolates with high cocoa-solid content. This will add further incentive to expand the production of fine or flavor Trinitario cocoa and promote commercialization activities based on it.

### 3.4. Cocoa-Rich Diet Improves Hepatic Lipid Metabolism in Zucker Diabetic Fatty Rats

#### Cordero-Herrera, I.; Martín, M.A.; Fernández-Millán, E.; Álvarez, C.; Goya, L.; Ramos S. *

Background and objectives: Diabetes is associated with altered lipid metabolism that could lead to ectopic-hepatic lipid deposition. Cocoa has been suggested to exert different biological activities contributing to prevent alterations of lipid metabolism as occurs in type 2 diabetes. The aim of the present study is to analyze the role of a cocoa-enriched diet in improving lipid metabolism in the liver of type 2 diabetic Zucker diabetic fatty (ZDF) rats.

Methodology: Male ZDF rats were fed a control or cocoa-rich diet (10%), and Zucker lean (ZL) animals received the control diet. Serum and hepatic lipid levels, protein kinase B (AKT), 5′-AMP-activated protein kinase (AMPK), protein kinase Cζ (PKCζ), fatty acid synthase (FAS), sterol regulatory element-binding protein 1 (SREBP-1) and proliferator-activated receptor α (PPARα) expression were evaluated.

Results: ZDF rats fed with cocoa decreased body weight gain and improved circulating and hepatic lipid levels, namely triglycerides, cholesterol and non-esterified fatty acids. The lipid-lowering effect was related to the diminished synthesis mediated by SREBP1-c and FAS and the increased fatty acid oxidation (values of PPARα). All these effects depend on AMPK, AKT and PKCζ, whose levels of phosphorylation were returned to control values upon cocoa administration in ZDF rats.

Conclusion: Cocoa improves lipid metabolism in ZDF rats through the modulation of multiple signalling pathways.

Acknowledgments: Grants AGL2010-17579, BFU2011-25420 and CIBERDEM from MICINN. I. Cordero-Herrera is a fellow of the FPI predoctoral program (MICINN).

### 3.5. Amplification of the nrf2 Neuroprotective Pathway via Potentially Low Brain Cocoa Flavanol Levels against Stroke in Young and also Aged Animals

#### Doré, S.

Pharmacological treatment approaches for stroke and other neurodegenerative diseases have been largely unsuccessful. Since ischemic stroke is predominantly a disease of the elderly, issues such as side effects, co-morbidities and contraindications are particularly problematic. Due to such health considerations, natural bioactive compounds are attractive alternatives to standard therapies. Studies suggest that the flavan-3-ol (–)-epicatechin (EC), which is lipophilic and has essentially no reported toxicity, can protect against cerebrovascular disease and stroke. Although various flavan-3-ols have shown efficacy in young, healthy preclinical animal models, the potential of achieving such benefits in aged mice is understudied. Additionally, the *in vivo* mechanisms of protection remain elusive. We hypothesized that EC confers its health benefits by activating the transcriptional factor Nrf2, which exerts pleiotropic effects by upregulating key cytoprotective proteins. Here, wild type control or Nrf2-/- mice aged four or 12 months were subjected to a permanent stroke model 90 min following oral administration of the minimum effective EC dose established in our previous stroke studies. Similar to previous results with young mice, 12-month-old wild types also showed significant reductions in infarct volume (41.0%) and improved performance in removing adhesive tape relative to vehicle-treated controls; whereas, such a significant beneficial effect was not observed in Nrf2-/-. However, EC did not reduce immunoreactivity for the microglia/macrophage marker anti-Iba1, suggesting that dampened activation/recruitment did not account for EC brain protection. Furthermore, there were no differences in mouse IgG extravasation or spontaneous hemorrhage between EC-treated groups. These findings further validate EC building brain resistance to oxidative stress not only in young but also in aging mice and suggest that EC prophylaxis may confer benefits, at least in part, by reducing brain hemorrhage.

### 3.6. Metabolic Impact of Cocoa Diet on the Urinary Metabolic Profiles of Rats

#### Massot-Cladera, M. *; Mayneris-Perxachs, J.; Swann, J.R.; Costabile, A.; Castell, M.; Pérez-Cano, F.J.

Background and Objectives: Cocoa contains fiber and polyphenols that influence the intestinal ecosystem by affecting the growth of certain species of rat’s gut microbiota (Massot-Cladera, M., *et al.* *Arch. Biochem. Biophys.* 2012, *527*, 105–112; Massot-Cladera, M., *et al.* *Br. J. Nutr.* 2014, *112*, 1944–1954; Massot-Cladera, M., *et al.* *J. Funct. Foods* 2015, *19*, 341–352. The aim of the present study was to ascertain whether cocoa also modulates bacterial metabolism and also to establish the particular involvement of cocoa fiber in such effects.

Methodology: For this purpose, Wistar rats were fed, for three weeks, a standard diet (REF), a diet containing 10% cocoa (providing a final proportion of 0.4% of polyphenols, 0.85% of soluble fiber and 2.55% of insoluble fiber) (C10), a diet with the same soluble and insoluble fiber proportion from cocoa as the C10 diet, but with a very low amount of polyphenols (<0.02%) (CF) or a reference fiber diet (I) containing 0.85% soluble fiber as inulin. After two weeks of intervention, rats were maintained in metabolic cages for 24 h to collect urine output. Urine was centrifuged and frozen at −80 °C until NMR analysis.

Results and Conclusions: There were no differences between the two reference diets (REF and I diets). Animals fed the C10 diet excreted higher levels of epicatechin than those from the I and CF groups. Although both the C10 (OPLSDA model Q2Y = 0.68) and the CF (Q2Y = 0.88) groups excreted more caffeine, theobromine, theophylline, paraxanthine, and malonate than the I group, the C10 diet group excreted higher amounts of these metabolites. Moreover, C10-fed animals excreted higher amounts of 2-oxoglutarate, citrate, choline, allantoin, *N*-acetylglycoprotein, hippurate, sucrose and glucose than the CF-fed animals. Due to the fact that the C10 diet had a higher impact on the urinary metabolic profile than the CF diet, it is likely that other cocoa compounds, rather than cocoa fiber, are responsible for these effects.

Acknowledgments: Grants AGL2008-02790 and AGL2011-24279 from the Spanish Ministry of Science and Innovation and the Ministry of Economy and Competitiveness, respectively.

### 3.7. Benefits of Dark Chocolate Ingestion over the Central Arterial Hemodynamics in Young Healthy People

#### Pereira, T. *; Conde, J.

Introduction: The aim of this study was to assess the vascular benefits of a dark chocolate intake program, particularly over the central arterial hemodynamics, in healthy and young individuals.

Methods: A randomized and controlled trial was carried out involving 60 healthy young individuals, randomized into two groups: control group (CG; *n* = 30) and intervention group (IG; *n* = 30). The IG ingested a daily dosage of 10 g of dark chocolate (>75% cocoa) for a month. All the individuals were submitted to two clinical evaluations, basal and after one month of intervention, in which their weight, height, body mass index (BMI), systolic blood pressure (SBP), diastolic blood pressure (DBP), heart rate (HR), arterial stiffness index (ASI), aortic pulse wave velocity (PWV) and pulse wave analysis over the carotid artery (PWA) were assessed.

Results: CG and IG groups had similar baseline clinical and demographic characteristics. After the intervention, BMI, HR and brachial BP did not suffer significant variations in either group. The baseline PWV and PWA parameters were similar in both groups, but were significantly different in the second evaluation, with the IG showing lower PWV, lower ASI and lower augmentation indexes (AiX). Arterial function improved after intervention in the IG, with PWV decreasing from 6.1 ± 0.41 m/s (baseline) to 5.83 ± 0.53 m/s (post-intervention; *p* = 0.02), with no significant differences observed in the CG. A significant decrease in ASI (0.16 ± 0.01 at baseline and 0.13 ± 0.01 post-intervention; *p* < 0.001) and AiX (at baseline −15.88 ± 10.75 and post-intervention −22.57 ± 11.16; *p* = 0.07) were also depicted for the IG, but not for the CG.

Conclusion: The daily ingestion of 10 g dark chocolate (>75% cocoa) for a month improves the vascular function in young and healthy individuals, increasing aortic distensibility and positively modulating the reflected waves.

### 3.8. The Regular Daily Intake of Dark Chocolate Improves the Endothelial Function in Young and Healthy Individuals

#### Pereira, T. *; Conde, J.

Introduction: The aim of this study was to assess the vascular benefits of a dark chocolate intake program, particularly for the endothelial function, in healthy and young individuals.

Methods: A randomized and controlled trial was carried out involving 60 healthy young individuals, randomized into two groups: control group (CG; *n* = 30) and intervention group (IG; *n* = 30). The IG ingested a daily dosage of 10 g of dark chocolate (>75% cocoa) for a month. All the individuals were submitted to two clinical evaluations, basal and after one month of intervention, in which their weight, height, body mass index (BMI), systolic blood pressure (SBP), diastolic blood pressure (DBP), heart rate (HR) and flow-mediated dilation (FMD) were assessed.

Results: CG and IG groups had similar baseline clinical and demographic characteristics. After the intervention, BMI, HR and brachial BP did not suffer significant variations in either group. The basal FMD was similar in the IG and the CG, but was significantly different in the second evaluation, with the IG showing higher FMD values (23.22% ± 7.64% *versus* 13.23% ± 5.76%, respectively for the IG and the CG; *p* < 0.001). Endothelial function improved in the IG, with the FMD increasing 9.31% after the one-month intervention (*p* < 0.001), with no significant variation in the CG.

Conclusion: The daily ingestion of 10 g dark chocolate (>75% cocoa) for a month has a positive modulation effect over the endothelium-dependent vasodilation in young and healthy individuals. This optimization of endothelial function could play a crucial role in cardiovascular preventive strategies, although further research is needed to clearly demonstrate the benefits of dark chocolate in the long term.

### 3.9. A Single Portion of Cocoa High in Flavanols Acutely Improves Microcirculation in Human Skin

#### Heinrich, U. *; Garbe, B.; Tronnier, H.; Stahl, W.

Background and objectives: Long-term ingestion of cocoa rich in flavanols increases cutaneous blood flow and improves skin condition in humans. Aim: To investigate the acute effects of a single dose of cocoa rich in flavanols on dermal microcirculation.

Methodology: In a crossover design study, 10 healthy women ingested a cocoa drink with high (329 mg) or low (27 mg) content of flavanols. The major flavanol monomer in both drinks was epicatechin, 61 mg in the high flavanol and 6.6 mg in the low flavanol product per 100 mL. Dermal blood flow and oxygen saturation were examined by laser Doppler flowmetry and spectroscopically at 1 mm skin depth at times = 0, 1, 2, 4 and 6 h. At the same time points, plasma levels of total epicatechin (free compound plus conjugates) were measured by means of HPLC (Heinrich, U., *et al.* Wilson, P.K., Hurst, W.J., Eds. *Choc. Heal. Chem. Nutr. Ther*. The Royal Society of Chemistry: Cambridge, UK, 2015; pp. 179–195).

Results and conclusion: Subsequent to the intake of high flavanol cocoa, dermal blood flow was significantly increased by 1.7-fold at t = 2 h and oxygen saturation was elevated 1.8-fold. No statistically significant changes were found upon intake of low flavanol cocoa. Maximum plasma levels of total epicatechin were observed 1 h after ingestion of the high flavanol cocoa drink, 11.6 ± 7.4 nmol/L at baseline, and 62.9 ± 35.8 nmol/L at 1 h. No change of total epicatechin was found in the low flavanol group. Flavanol-rich cocoa acutely improves dermal blood flow and oxygen saturation. Dietary flavanols may contribute to the maintenance of skin health and may influence skin appearance.

### 3.10. Development and Validation of a Food Frequency Questionnaire to Assess Cocoa Consumption in University Students

#### Vicente, F. *; Saldaña-Ruíz, S.; Rabanal, M.; Rodríguez-Lagunas, M.J.; Pereira, P.; Pérez-Cano, F.J.; Castell, M.

Introduction: Although cocoa has been recognized as a valuable source of polyphenols, epidemiological studies show it makes a low contribution to total polyphenol intake. These results could be due to the lack of thorough Food Frequency Questionnaires (FFQ) intended to assess the consumption of cocoa/chocolate products, thus its contribution may well be underestimated.

Objective: The aim of the present work was to validate a FFQ designed to assess cocoa consumption in a prone population—university students.

Methods: A sample of 50 students was recruited to complete the developed 90-item questionnaire (C-FFQ), a validated questionnaire about chocolate and energy drink intake (EFSA-Q) as well as a 24-h dietary recall (24HDR). To study agreement between the C-FFQ and the EFSA-Q/24HDR, the correlation between data (Spearman’s test) and the comparison of consumption frequency data (Wilcoxon test) were calculated. In addition, Bland Altman and the quintile classification analyses were performed.

Results: Significant correlations between the C-FFQ and the EFSA-Q/24HDR for the most common cocoa and chocolate products were observed. A large variety of cocoa/chocolate products frequently consumed by the participants were detected by the C-FFQ and 24HDR, whereas they were not included in the EFSA-Q. When considering percentile classification, the majority of the individuals were classified in the same/adjacent quintile for the C-FFQ and the EFSA-Q or 24HDR.

Conclusion: In conclusion, the developed 90-item FFQ can be considered as a valid option for assessing the consumption frequency of cocoa- and chocolate-derived products. As far as we know, this is the most extensive questionnaire developed in relation to a wide range of several products containing chocolate in a traditional European diet. The use of this FFQ will allow individuals to be reasonably classified according to their cocoa consumption and therefore an evaluation of the impact of such intake on health and lifestyle in further studies.

### 3.11. Cocoa Intake Alleviates Hepatic Oxidative Stress in Zucker Diabetic Fatty Rats

#### Cordero-Herrera, I.; Martín, M.A.; Goya, L.; Ramos, S. *

Background and objectives: Chronic hyperglycemia in diabetes is associated with oxidative stress-mediated tissue damage. Cocoa and dark chocolate have demonstrated the ability to improve insulin sensitivity and modulate oxidative stress markers in diabetic patients. The present study is aimed at exploring the role of a cocoa-enriched diet in ameliorating the oxidative stress-induced damage in the liver of type 2 diabetic Zucker diabetic fatty (ZDF) rats.

Methodology: Male ZDF rats were fed a control or cocoa-rich diet (10%), and Zucker lean (ZL) animals received the control diet. Hepatic reactive oxygen species (ROS), carbonyl and glutathione (GSH) levels, as well as superoxide dismutase (SOD), heme oxygenase (HO-1), glutathione-S-transferase (GST), glutathione peroxidase (GPx), glutathione reductase (GR) and catalase (CAT) activities, and nuclear factor erythroid-derived 2-like 2 (Nrf2), as well as p65-nuclear factor-kappaB (NF-κB) expression were evaluated.

Results: Cocoa diet reduced ROS levels and carbonyl content in the liver of ZDF animals. The diminished activity of SOD and the enhanced activity of HO-1 in ZDF-C were returned to ZL values upon cocoa administration. Cocoa did not restore the decreased GST activity in either ZDF groups in comparison to ZL rats. GSH content and activities of GPx, GR and CAT remained unaltered among all animal groups. Moreover, the cocoa-rich diet suppressed total and phosphorylated Nrf2, as well as p65-NF-κB-enhanced levels observed in ZDF rats.

Conclusion: Cocoa protects the hepatocytes by improving the antioxidant competence in the liver of young type 2 diabetic ZDF rats.

Acknowledgments: Grants AGL2010-17579, and CIBERDEM from MICINN. I. Cordero-Herrera is a fellow of the FPI predoctoral program (MICINN).

### 3.12. Gut Microbiota and Mucosal Immunity Are Influenced by Cocoa the Obromine

#### Martín-Peláez, S. *; Rigo-Adrover, M.; Camps-Bossacoma, M.; Massot-Cladera, M.; Saldaña-Ruiz, S.; Franch, A.; Pérez-Cano, F.J.; Castell, M.

Background and objectives: Ingestion of cocoa has been shown to influence the immune system. The role of the bioactive components of cocoa (*i.e.*, flavonoids, fiber, theobromine) is not completely understood. The objective of this study was to evaluate the effect of cocoa theobromine on the intestinal compartment.

Methodology: Twenty-one three-week-old Lewis rats, were randomly assigned to three dietary groups (*n* = 7 per group) for 15 days: the reference group (RF), which was fed with a standard diet; the cocoa group (CC), which was fed with a standard diet containing 10% of a conventional cocoa powder; and the theobromine group (TB), which was fed with a standard diet including 0.25% theobromine (the same amount of theobromine as the CC diet). Fecal samples were obtained at day 15 in order to establish bacterial populations, short chain fatty acids (SCFA), pH and IgA coating bacteria.

Results: The TB diet significantly inhibited the growth of the bacterial groups covered by *E. coli*, *Bif164*, *Strept* and *Chis150* probes compared to RF. This inhibitory effect was not observed when theobromine was ingested with cocoa (CC diet), except for *E. coli*. Both CC and TB diets increased the fecal amounts of total SCFA, but led to different percentages of the individual SCFA analyzed, except for butyric acid, which increased with both diets. The TB increased percentages of formic acid, whereas the CC diet increased the percentage of acetic acid. Only the TB diet led to changes in fecal pH, which was found to be significantly higher than the RF and CC diets. The TB and in particular the CC diets significantly decreased the percentages of fecal IgA-coating bacteria compared to the RF diet.

Conclusions: Theobromine present in cocoa has an important role in rat’s intestinal gut microbiota and immune system modulation.

Acknowledgments: Grant AGL2011-24279 from the Spanish Ministry of Economy and Competitiveness.

### 3.13. Nutrimetabolomic Approach to Identify Biomarkers for Cocoa-Food Products Consumption Monitoring in Healthy Volunteers

#### Llorach, R. *; Farran-Codina, A.; Soriano, A.; Termes-Escalé, M.; Luna, O.; Martinez, C.; Bosch, N.; Garrido, P.; Llobet, J.M.; Andres-Lacueva, C.; Urpi-Sarda, M.

Background and objectives: Cocoa consumption has been related with some health-promoting activities mainly related to cardiovascular disease. Nutrimetabolomics explore the complex relationship between the consumption of dietary compounds and the maintenance of health or disease development with the aim of discovering new biomarkers of intake and effect. In this regard, the aim of this work was to study the urinary cocoa products’ fingerprint in order to establish a definition for the future for a specific biomarker imprinting of cocoa in healthy and young volunteers.

Methodology: Cocoa-food product intake was defined according to an FFQ that had been previously completed by free-living, healthy volunteers. Subjects were stratified as higher consumer (≥5 g/day), medium (between 1.16 and 4.28 g/day) and lower consumer (≤1 g/day). Urine samples of subjects were analyzed using HPLC-Q-TOF-MS, followed by multivariate data analysis (orthogonal signal correction for partial least squares-based classification/discrimination model or OSC-PLSDA and hierarchical cluster analysis or HCA). The metabolomics analysis was carried out using R package MAIT, which includes an in-house food metabolome database.

Results and conclusions: Urinary metabolome showed significant differences between the three consumer groups. Several metabolites were associated with the consumption of cocoa-foods, the most important being those coming from theobromine metabolism. In addition, microbial polyphenol metabolites and vanillin-derived metabolites have also been putatively identified. These metabolites have been previously associated with cocoa intake in other populations; however, as far we know, this is one of the first time that these metabolites have been identified in a free-living, healthy and young population using a metabolomics approach. The study reinforces the interest in the replication of results as well as the capacity of metabolomics to identify cocoa-foods products’ footprint, combining epidemiological nutritional data and metabolomics.

### 3.14. Intestinal Evaluation of Cocoa Diet as an Antiallergic Compound in Food Allergic Rats

#### Abril-Gil, M. *; Pérez-Cano, F.J.; Saldaña-Ruiz, S.; Franch, A.; Castell, M.

Background and Objectives: Food allergy (FA) is a chronic disease that is mainly mediated by IgE against food allergen (Fox, M., *et al.* *Eur. J. Public Health* 2013, *23*, 757–762). Previous studies have demonstrated that a diet containing cocoa can attenuate the synthesis of IgE in a rat allergy model (Abril-Gil, M., *et al.* *Pharmacol Res* 2012, 65, 603–608). The aim of the present study was to study in depth the role of the cocoa diet in the intestine after the induction of an anaphylactic response (AR) in an FA model.

Methods: Brown Norway rats, sensitized with ovalbumin plus *Bordetella pertussis* toxin, were fed either a standard diet (FA-R group) or a diet containing 10% cocoa (FA-C group). Later, animals were orally given ovalbumin to develop FA. At Day 27, an AR was induced. Blood samples were collected to determine the hematocrit and intestinal barrier integrity (assessed by β-lactoglobulin administration). Finally, a small intestine piece was obtained for real-time PCR gene expression analysis.

Results and Conclusions: After AR induction, both FA-R and FA-C dies raised the hematocrit but animals from the FA-C group had an earlier recovery. The intestinal permeability was increased in both FA groups. Intestinal gene expression of the mast cell infiltration markers (FcεRI and protease-II) and TGF-β were not modified by FA, whereas they tended to decrease after the cocoa diet. In addition, the FA-R group reduced interleukin-10 expression, which was not detected in FA-C animals. In summary, cocoa diet in rats did not increase intestinal permeability in AR but attenuated mast cell infiltration and degranulation.

Acknowledgments: Grants AGL2008-02790 and AGL2011-24279 from the Spanish Ministry of Science and Innovation and Economy and Competitiveness, respectively.

## Appendix

### Author’s Affiliations

- Abril-Gil, M. Department of Physiology, Faculty of Pharmacy, University of Barcelona, Barcelon, Spain; Nutrition and Food Safety Research Institute (INSA-UB), Barcelona, Spain
- Álvarez, C. Centro de Investigación Biomédica en Red de Diabetes y Enfermedades Metabólicas Asociadas (CIBERDEM), ISCIII, Madrid, Spain; Departament of Biochemistry and Molecular Biology, Faculty of Pharmacy, Complutense University of Madrid, Madrid, Spain
- Andres-Lacueva, C. Biomarkers and Nutrimetabolomic Laboratory, Nutrition and Food Science Department, XaRTA, INSA, Food and Nutrition Torribera Campus, Pharmacy Faculty, University of Barcelona, Santa Coloma de Gramenet, Spain
- Bekele, F. Cocoa Research Centre, University of the West Indies, St. Augustine, Trinidad and Tobago
- Bitlloch-Obiols, M. Department of Physiology, Faculty of Pharmacy, University of Barcelona, Barcelona, Spain
- Bosch, N. Research Group on Physical Activity and Health, Nutrition and Food Science Department, Food and Nutrition Torribera Campus, Pharmacy Faculty, University of Barcelona, Santa Coloma de Gramenet, Spain
- Calvet, T. Crystallography Department, Barcelona University, Barcelona, Spain
- Camps-Bossacoma, M. Department of Physiology, Faculty of Pharmacy, University of Barcelona, Barcelona, Spain; Nutrition and Food Safety Research Institute (INSA-UB), Barcelona, Spain
- Castell, M. Department of Physiology, Faculty of Pharmacy, University of Barcelona, Barcelona, Spain; Nutrition and Food Safety Research Institute (INSA-UB), Barcelona, Spain
- Yen, I.C. Department of Chemistry, University of the West Indies, St. Augustine, Trinidad and Tobago
- Conde, J. Escola Superior de Tecnologia da Saúde de Coimbra, Coimbra, Portugal
- Cordero-Herrera, I. Department of Metabolism and Nutrition, Institute of Food Science, Technology and Nutrition (ICTAN-CSIC), Madrid, Spain
- Costabile, A. Department of Food and Nutritional Sciences, School of Chemistry, Food and Pharmacy, University of Reading, Reading, UK
- Cuevas-Diarte, M.A. Crystallography Department, Barcelona University, Barcelona, Spain
- Decroix, L. Department of Human Physiology, Faculty of Physical Education and Physical Therapy, Vrije Universiteit Brussel, Brussels, Belgium
- Dias-Soares, D. Department of Physical Education, University of Minas Gerais, Belo Horizonte, Brazil
- Doré, S. Departments of Anesthesiology, Neurology, Psychiatry, Psychology, Pharmaceutics and Neuroscience, Center for Translational Research in Neurodegenerative Disease, University of Florida, Gainesville, FL, USA
- Estruch, R. Department of Internal Medicine, Hospital Clinic, IDIBAPS, University of Barcelona, Barcelona, Spain; CIBER–Obesity and Nutrition, Health Institute “Carlos III”, Madrid, Spain
- Farran-Codina, A. Research Group on Physical Activity and Health, Nutrition and Food Science Department, Food and Nutrition Torribera Campus, Pharmacy Faculty, University of Barcelona, Santa Coloma de Gramenet, Spain
- Fernández-Millán, E. Centro de Investigación Biomédica en Red de Diabetes y Enfermedades Metabólicas Asociadas (CIBERDEM), ISCIII, Madrid, Spain
- Franch, A. Department of Physiology, Faculty of Pharmacy, University of Barcelona, Barcelona, Spain; Nutrition and Food Safety Research Institute (INSA-UB), Barcelona, Spain
- Franke, A.A. Epidemiology Program, University of Hawaii Cancer Center, Honolulu, HI, USA
- Garbe, B. DermaTronnier, Institute for Experimental Dermatology, Witten-Herdecke-University, Witten, Germany
- Garcia-Aloy, M. Biomarkers and Nutrimetabolomic Laboratory, Nutrition and Food Science Department, XaRTA, INSA, Food and Nutrition Torribera Campus, Pharmacy Faculty, University of Barcelona, Santa Coloma de Gramenet, Spain
- Garrido, P. Research Group on Physical Activity and Health, Nutrition and Food Science Department, Food and Nutrition Torribera Campus, Pharmacy Faculty, University of Barcelona, Santa Coloma de Gramenet, Spain
- Goya, L. Department of Metabolism and Nutrition, Institute of Food Science, Technology and Nutrition (ICTAN-CSIC), Madrid, Spain
- Heinrich, U. DermaTronnier, Institute for Experimental Dermatology, Witten-Herdecke-University, Witten, Germany
- Heyman, E. Department of Physical Activity, Muscle and Health, Faculté de Sciences du Sport et de l’Education Physique, Université de Droit et de la Santé Lille 2, Lille, France
- Jáuregui, O. Scientific and Technological Centers of the University of Barcelona (CCIT-UB), Barcelona, Spain
- Kolonel, L.N. Epidemiology Program, University of Hawaii Cancer Center, Honolulu, HI, USA
- Lambert, J.D. Department of Food Science and Center for Molecular Toxicology and Carcinogenesis, The Pennsylvania State University, University Park, PA, USA
- Le Marchand, L. Epidemiology Program, University of Hawaii Cancer Center, Honolulu, HI, USA
- Llobet, J.M. GRET-CERETOX, Pharmacology and Therapeutical Chemistry Department, School of Pharmacy, University of Barcelona, Barcelona, Spain; Nutrition and Food Safety Research Institute (INSA), Barcelona, Spain
- Llorach, R. Biomarkers and Nutrimetabolomic Laboratory, Nutrition and Food Science Department, XaRTA, INSA, Food and Nutrition Torribera Campus, Pharmacy Faculty, University of Barcelona, Santa Coloma de Gramenet, Spain
- Luna, O. Biomarkers and Nutrimetabolomic Laboratory, Nutrition and Food Science Department, XaRTA, INSA, Food and Nutrition Torribera Campus, Pharmacy Faculty, University of Barcelona, Santa Coloma de Gramenet, Spain
- Mans, C. Faculty of Chemistry, University of Barcelona, Barcelona, Spain
- Martín, M.A. Department of Metabolism and Nutrition, Institute of Food Science, Technology and Nutrition (ICTAN-CSIC), Madrid, Spain; Centro de Investigación Biomédica en Red de Diabetes y Enfermedades Metabólicas Asociadas (CIBERDEM), ISCIII, Madrid, Spain
- Martinez, C. Biomarkers and Nutrimetabolomic Laboratory, Nutrition and Food Science Department, XaRTA, INSA, Food and Nutrition Torribera Campus, Pharmacy Faculty, University of Barcelona, Santa Coloma de Gramenet, Spain
- Martín-Peláez, S. Department of Physiology, Faculty of Pharmacy, University of Barcelona, Barcelona, Spain; Cardiovascular Risk and Nutrition Research Group, Hospital del Mar Research Institute (IMIM), CIBER of Physiology of Obesity and Nutrition (CIBEROBN), Institute of Health Carlos III, Madrid, Spain
- Maskarinec, G. Epidemiology Program, University of Hawaii Cancer Center, Honolulu, HI, USA
- Massot-Cladera, M. Department of Physiology, Faculty of Pharmacy, University of Barcelona, Barcelona, Spain; Nutrition and Food Safety Research Institute (INSA-UB), Barcelona, Spain
- Mayneris-Perxachs, J. Department of Food and Nutritional Sciences, School of Chemistry, Food and Pharmacy, University of Reading, Reading, UK
- Meeusen, R. Department of Human Physiology, Faculty of Physical Education and Physical Therapy, Vrije Universiteit Brussel, Brussels, Belgium; School of Public Health, Tropical Medicine and Rehabilitation Sciences, James Cook University, Queensland, Australia
- Milenković, M. Department of Microbiology and Immunology, Faculty of Pharmacy, Belgrade University, Belgrade, Serbia
- Monroe, K.R. Department of Preventive Medicine, University of Southern California, Los Angeles, CA, USA
- Morimoto, Y. Epidemiology Program, University of Hawaii Cancer Center, Honolulu, HI, USA
- Pereira, P. Instituto Superior de Ciências da Saúde Egas Moniz, Egas Moniz Cooperativa de Ensino Superior, Monte da Caparica, Portugal
- Pereira, T. Escola Superior de Tecnologia da Saúde de Coimbra, Coimbra, Portugal
- Pérez-Cano, F.J. Department of Physiology, Faculty of Pharmacy, University of Barcelona, Barcelona, Spain; Nutrition and Food Safety Research Institute (INSA-UB), Barcelona, Spain
- Pilgrim, S. Department of Chemistry, University of the West Indies, St. Augustine, Trinidad and Tobago
- Rabanal, M. Department of Physiology, Faculty of Pharmacy, University of Barcelona, Barcelona, Spain; Nutrition and Food Safety Research Institute (INSA-UB), Barcelona, Spain
- Ramos, S. Department of Metabolism and Nutrition, Institute of Food Science, Technology and Nutrition (ICTAN-CSIC), Madrid, Spain
- Rigo-Adrover, M. Department of Physiology, Faculty of Pharmacy, University of Barcelona, Barcelona, Spain; Nutrition and Food Safety Research Institute (INSA-UB), Barcelona, Spain
- Rodríguez-Lagunas, M.J. Department of Physiological Sciences II, Faculty of Medicine, University of Barcelona, Barcelona, Spain; Nutrition and Food Safety Research Institute (INSA-UB), Barcelona, Spain
- Rovira, E. Enric Rovira, SL Castellbell i el Vilar, Spain
- Saldaña-Ruíz, S. Department of Physiology, Faculty of Pharmacy, University of Barcelona, Barcelona, Spain; Nutrition and Food Safety Research Institute (INSA-UB), Barcelona, Spain
- Sato, K. Hiroshima University, Higashi-Hiroshima, Japan
- Šoajić, S. Department of Bromatology, Faculty of Pharmacy, Belgrade University, Belgrade, Serbia
- Soriano, A. Biomarkers and Nutrimetabolomic Laboratory, Nutrition and Food Science Department, XaRTA, INSA, Food and Nutrition Torribera Campus, Pharmacy Faculty, University of Barcelona, Santa Coloma de Gramenet, Spain
- Stahl, W. Institute for Biochemistry & Molecular Biology I, Heinrich-Heine-University, Duesseldorf, Germany
- Sukha, D. Cocoa Research Centre, University of the West Indies, St. Augustine, Trinidad and Tobago
- Swann, J.R. Department of Food and Nutritional Sciences, School of Chemistry, Food and Pharmacy, University of Reading, Reading, UK
- Tagougui, S. Department of Physical Activity, Muscle and Health, Faculté de Sciences du Sport et de l’Education Physique, Université de Droit et de la Santé Lille 2, Lille, France
- Termes-Escalé, M. Biomarkers and Nutrimetabolomic Laboratory, Nutrition and Food Science Department, XaRTA, INSA, Food and Nutrition Torribera Campus, Pharmacy Faculty, University of Barcelona, Santa Coloma de Gramenet, Spain
- Todorović, V. Department of Bromatology, Faculty of Pharmacy, Belgrade University, Belgrade, Serbia
- Tomás-Barberán, F.A. Research Group on Quality, Safety and Bioactivity of Plant Foods, Department of Food Science and Technology, CEBAS-CSIC, Murcia, Spain
- Tonoli, C. Department of Human Physiology, Faculty of Physical Education and Physical Therapy, Vrije Universiteit Brussel, Brussels, Belgium; Department of Physical Activity, Muscle and Health, Faculté de Sciences du Sport et de l’Education Physique, Université de Droit et de la Santé Lille 2, Lille, France
- Tresserras, J. Department of Prehistory, Ancient History and Archaeology, University of Barcelona, Barcelona, Spain
- Tronnier, H. DermaTronnier, Institute for Experimental Dermatology, Witten-Herdecke-University, Witten, Germany
- Ueno, S. Hiroshima University, Higashi-Hiroshima, Japan
- Urpi-Sarda, M. Biomarkers and Nutrimetabolomic Laboratory, Nutrition and Food Science Department, XaRTA, INSA, Food and Nutrition Torribera Campus, Pharmacy Faculty, University of Barcelona, Santa Coloma de Gramenet, Spain
- Vauzour, D. Norwich Medical School, James Watson Road, University of East Anglia, Norwich Research Park, Norwich, UK
- Vázquez-Fresno, R. Biomarkers and Nutrimetabolomic Laboratory, Nutrition and Food Science Department, XaRTA, INSA, Food and Nutrition Torribera Campus, Pharmacy Faculty, University of Barcelona, Santa Coloma de Gramenet, Spain
- Verhagen, H. National Institute for Public Health and the Environment (RIVM), Bilthoven, The Netherlands
- Vicente, F. Department of Physiology, Faculty of Pharmacy, University of Barcelona, Barcelona, Spain; Instituto Superior de Ciências da Saúde Egas Moniz, Egas Moniz Cooperativa de Ensino Superior, Monte da Caparica, Portugal
- Wilkens, L.R. Epidemiology Program, University of Hawaii Cancer Center, Honolulu, HI, USA

